# Systematic Pan-Cancer Analysis Identifies PHF6 as an Immunological and Prognostic Biomarker

**DOI:** 10.3390/diagnostics16010110

**Published:** 2025-12-29

**Authors:** Yi Tao, Mingming Hu, Zixin Guo, Qian Xu, Lu Niu, Yonghong Mao, Gang Yuan

**Affiliations:** Department of Thoracic Surgery, Institute of Thoracic Oncology, Frontiers Science Center for Disease-Related Molecular Network, West China Hospital, Sichuan University, Chengdu 610041, China; taoyii@stu.scu.edu.cn (Y.T.); hu18712132276@foxmail.com (M.H.);

**Keywords:** PHF6, pan-cancer, tumor microenvironment, immune infiltration, prognosis

## Abstract

**Background/Objectives**: PHF6 is a chromatin-binding protein located in the nucleus, and it is involved in transcriptional regulation. However, limited research exists on the specific roles and mechanisms of PHF6 across various tumors. **Methods**: Based on The Cancer Genome Atlas (TCGA) database, we analyzed PHF6 expression in pan-cancer. We first evaluated the relevance between PHF6 and prognosis; then, the relevance between PHF6 and immune cell infiltration in pan-cancer were analyzed. Subsequently, we explored the correlation between PHF6 and cancer heterogeneity, such as tumor mutation burden (TMB) and microsatellite instability (MSI), as well as cancer stemness. Finally, the role of PHF6 was validated in liver cancer and pancreatic cancer cell lines by cell proliferation assays. **Results**: PHF6 expression was higher in the vast majority of cancers than their normal counterparts. PHF6 was substantially correlated with prognosis and immune cell infiltration in various cancers. Moreover, PHF6 expression showed a strong correlation with cancer heterogeneity and stemness in certain cancer types. Additionally, the depletion of PHF6 inhibited cell proliferation in both liver and pancreatic cancer cells. **Conclusions**: PHF6 expression was closely associated with the occurrence and development of many types of cancer, and it might promote cancer progression by inhibiting the function of the immune microenvironment, while knockout of PHF6 significantly inhibited the tumor cells proliferation.

## 1. Introduction

PHF6 is a crucial epigenetic regulator located on the X chromosome, and it is highly conserved across different species [1]. PHF6 encodes a protein with two PHD zinc finger domains capable of binding target DNA to regulate gene expression [2]. The PHD domain serves a dual role in DNA binding and chromatin remodeling. PHF6 has been reported to regulate cellular functions through various mechanisms. PHF6 interacts with the nucleosome deacetylation complex (NuRD) in the physical structure, controls nucleosome localization and DNA transcription, and plays an important regulatory role in genome integrity and cell cycle progression [3]. PHF6 is involved in DNA damage repair. PHF6 is rapidly recruited to the damage sites and facilitates classical non-homologous end connection (NHEJ) for DNA repair through the PARP pathway [4,5]. In addition, PHF6 directly participates in the process of transcription, interacting with transcription initiation complex activator UBF and the transcription elongation complex PAF1 [6,7].

Mutations can occur at various sites within the entire protein structure of PHF6, with most leading to protein inactivation. Mutations in the PHF6 gene can lead to Börjeson–Forssman–Lehmann Syndrome (BLFS). Additionally, PHF6 mutations are present in up to 30% of T-cell acute lymphoblastic leukemia (T-ALL) cases, 3% of acute myeloid leukemia (AML) cases, and some other forms of acute leukemia [8,9]. However, PHF6 is rarely studied in other cancer types. Therefore, we analyzed the expression and potential biological function of PHF6 in pan-cancer and validated its biological function in liver cancer and pancreatic cancer cells.

## 2. Materials and Methods

### 2.1. Data Resources

The TCGA Pan-Cancer (PANCAN, *N* = 10,535) and TCGA TARGET GTEx (PANCAN, *N* = 19,131) standardized datasets were obtained from UCSC (https://xenabrowser.net/ (accessed on 14 January 2025)). The expression data was normalized to TPM from raw counts to correct length/depth biases. Batch effects were removed via ComBat-seq/ComBat. The expression data of PHF6 were extracted from various samples, with log2 (x + 1) transformation. Cancer types with fewer than three samples were excluded.

### 2.2. PHF6 Expression Analysis

The unpaired Wilcoxon test was employed to compare expression differences between normal and tumor samples. Immunohistochemistry results for PHF6 in both cancerous and normal tissues were retrieved from the Human Protein Atlas (HPA) database [10].

### 2.3. Survival Analysis

The Cox regression model was employed to evaluate the correlation between PHF6 with prognosis in the TCGA Pan-Cancer dataset. The likelihood test was utilized to evaluate the prognostic significance. The results were presented as forest plots and survival curves.

### 2.4. Immune Infiltration and Enrichment Analysis

Using the TCGA Pan-Cancer dataset, the stromal score, immune score, and ESTIMATE score was calculated for each sample by the “ESTIMATE” algorithm [11]. Immune cell infiltration scores were assessed based on gene expression using the “CIBERSORT” algorithm [12]. Spearman’s analysis was conducted to calculate the relationship between PHF6 and immune infiltration scores, utilizing the “psych” R package (version 2.5.3).

### 2.5. Enrichment Analysis

In GEPIA2 (http://gepia2.cancer-pku.cn/ (accessed on 17 September 2025)), we utilized the “Similar Genes” module to identify genes significantly correlated with PHF6 across the pan-cancer dataset. Gene expression pattern similarity was quantified using the Pearson correlation coefficient (PCC), and genes were ranked in descending order of PCC values. The top 100 genes with the strongest correlations were selected for functional enrichment analysis, aiming to elucidate the biological functions associated with PHF6. Gene Ontology (GO) analysis and Kyoto Encyclopedia of Genes and Genomes (KEGG) analysis were performed on PHF6-related genes using the R package “clusterProfiler” (version 4.16.0), with a *p*-value of less than 0.05 considered significant.

### 2.6. Drug Sensitivity Analysis

The R package “oncoPredict” (version 1.2) was used to estimate the IC50 values of the samples, based on the Genomics of Drug Sensitivity in Cancer (GDSC) [13] (https://www.cancerrxgene.org/ (accessed on 9 December 2024)), to assess the sensitivity differences between the high and low expression groups of PHF6. oncoPredict trained regularization models on GDSC cell line gene expression and drug IC_50_, mapped feature-matched TCGA tumor expression to GDSC drug response via IC_50_ prediction, and used tumor-type stratification to boost reliability.

### 2.7. Correlation Analysis

The expression of immune checkpoint genes was obtained for correlation analysis. Tumor mutation burden (TMB) is highly correlated with the efficacy of PD-1/PD-L1 inhibitors, which enables some cancer patients to predict the effectiveness of immunotherapy through TMB markers [14]. Microsatellite instability (MSI) results from functional defects in DNA mismatch repair in tumor tissues, which is an important tumor marker in clinical settings [15]. Cancer stem cells are key regulators of tumor progression. DNAss assesses tumor stemness based on DNA methylation level, while RNAss assesses tumor stemness based on RNA expression [16]. The expression levels of RNA m1A modification genes were obtained for correlation analysis.

### 2.8. Cell Culture and Transfection

Liver cancer cell lines (HepG2, HuH-7), HEK-293T, and PAAD cell lines (CFPAC-1, MIA-PaCa-2) were obtained from the Stem Cell Bank of the Chinese Academy of Sciences in Shanghai, China. All cell lines were incubated at 37 °C with 5% CO_2_. For CRISPR–Cas9 knockout, virus particles were produced by co-transfecting HEK-293T cells with the lentiCRISPRv2/puro (Addgene) construct expressing the specific guide RNAs, psPAX2, and pMD2.G in a 4:3:1 mass ratio. The guide RNA oligo sequences used were the following: sgControl:CTTCGAAATGTCCGTTCGGT, sgPHF6#1: GGCAGCGCACCATAAGTGCA, and sgPHF6#2:AGTGACACCAGGCCTAAATG. Forty-eight hours post-transfection, target cells were transduced with 0.45 μm filtered viral supernatant, followed by selection with 2 μg/mL puromycin 24 h after media replacement.

### 2.9. Cell Proliferation Assay

Cell viability was assessed at 0, 1, 2, 3, 4, and 5 days using the CCK-8 assay kit (Yeasen, Shanghai, China). Cells were seeded at 2000 cells per well in 96-well plates, and after a 2 h incubation with CCK-8 reagent, absorbance at 450 nm was measured. The transfected cells were seeded into the 6-well plate (HepG2 cells: 1000 cells per well; all other cell lines: 2000 cells per well) for colony formation assay. The cells were fixed with 4% paraformaldehyde and stained with crystal violet when distinct cell colonies became visible. All experiments were performed in triplicate.

### 2.10. Statistical Analysis

The Wilcoxon test was employed to compare the expression differences. Student’s t-test and ANOVA were utilized for comparisons in the CCK-8 and colony formation assay. Spearman’s correlation analysis measured the correlation degree between specific variables. The statistical significance was defined as *p* < 0.05. The presentation of some charts and graphs was achieved by utilizing the Sangerbox tool within the R project [17].

## 3. Results

### 3.1. Expression of PHF6 in Pan-Cancer

**Figure 1:**
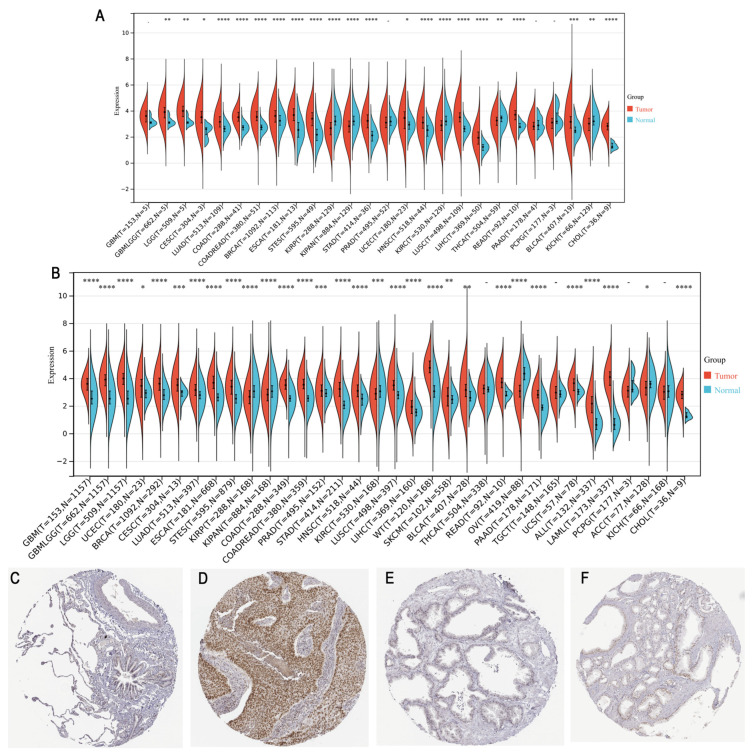
Analysis of PHF6 expression across cancer types. (**A**) PHF6 expression levels in different types of cancer in the TCGA database. (**B**) PHF6 expression levels in combination with the GTEx and TCGA databases. * *p* < 0.05, ** *p* < 0.01, *** *p* < 0.001, **** *p* < 0.0001. (**C**–**F**) IHC images of PHF6 expression in normal lung (**C**), lung cancer (**D**), prostate (**E**), and prostate cancer (**F**) from the HPA database [10]. Magnification: 20**×**.

PHF6 expression was upregulated in most tumor tissues compared with normal tissues, except for kidney cancer and thyroid carcinoma (THCA) (Figure 1A). Similarly, using data from the TCGA and GTEx databases, PHF6 expression was significantly higher in most cancer types, except for kidney cancer, ovarian serous cystadenocarcinoma (OV), and adrenocortical carcinoma (ACC) (Figure 1B). Immunohistochemical results obtained from the HPA database indicated that PHF6 protein levels in lung cancer (Figure 1C,D) and prostate cancer (Figure 1E,F) were higher than that in normal tissues.

### 3.2. Prognostic Value of PHF6 in Pan-Cancer

**Figure 2:**
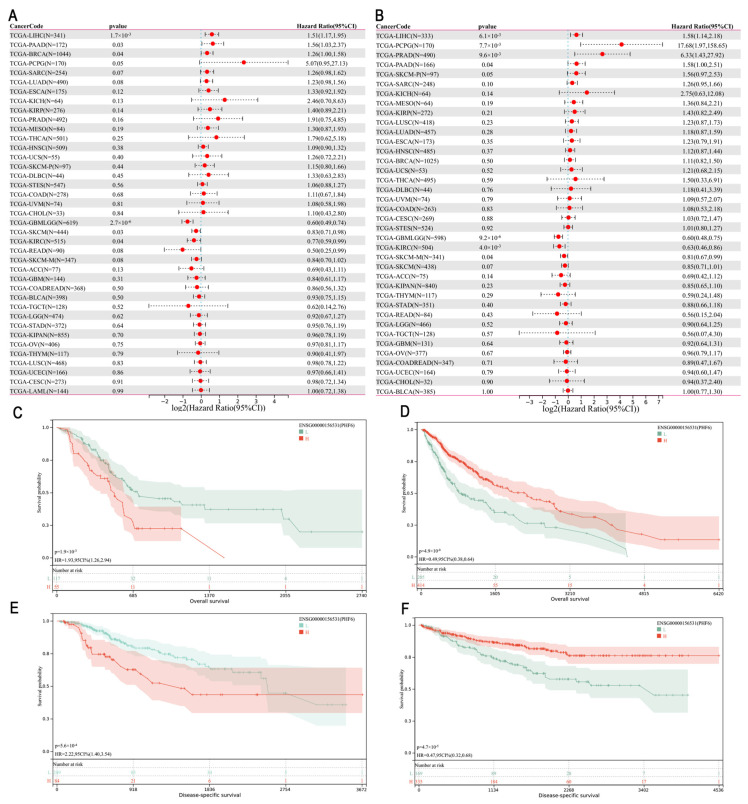
Prognostic significance of PHF6 in the TCGA dataset. (**A**) The association between PHF6 expression and OS. (**B**) The association between PHF6 expression and DSS. (**C**,**D**) The association between PHF6 and OS in PAAD (**C**) and GBMLGG cohorts (**D**). (**E**,**F**) The association between PHF6 and DSS in LIHC (**E**) and KIRC cohorts (**F**).

We employed univariate Cox regression analysis to examine the association between PHF6 levels and prognosis across various cancers to evaluate the prognostic role of PHF6. A forest plot was employed to depict the relationship of PHF6 with overall survival (OS) and disease-specific survival (DSS) (Figure 2A,B) in different cancer types. PHF6 was identified as an adverse factor for OS in liver hepatocellular carcinoma (LIHC), pancreatic adenocarcinoma (PAAD), and breast invasive carcinoma (BRCA) (Figure 2A). Furthermore, PHF6 was found to be a prognostic disadvantage factor for DSS in LIHC, pheochromocytoma and paraganglioma (PCPG), prostate adenocarcinoma (PRAD), and PAAD (Figure 2B). Conversely, PHF6 served as a favorable prognostic factor for both DSS and OS in glioma (GBMLGG), skin cutaneous melanoma (SKCM), and kidney renal clear cell carcinoma (KIRC) (Figure 2A,B). The survival curves demonstrated the correlation between PHF6 and the OS in PAAD (Figure 2C) and glioma (Figure 2D), which aligned with the trends observed in the forest plot. Additionally, in the DSS curve, PHF6 was a poor prognostic factor in LIHC (Figure 2E) but a favorable prognostic factor in KIRC (Figure 2F).

### 3.3. Immune Infiltration and Enrichment Analysis

**Figure 3:**
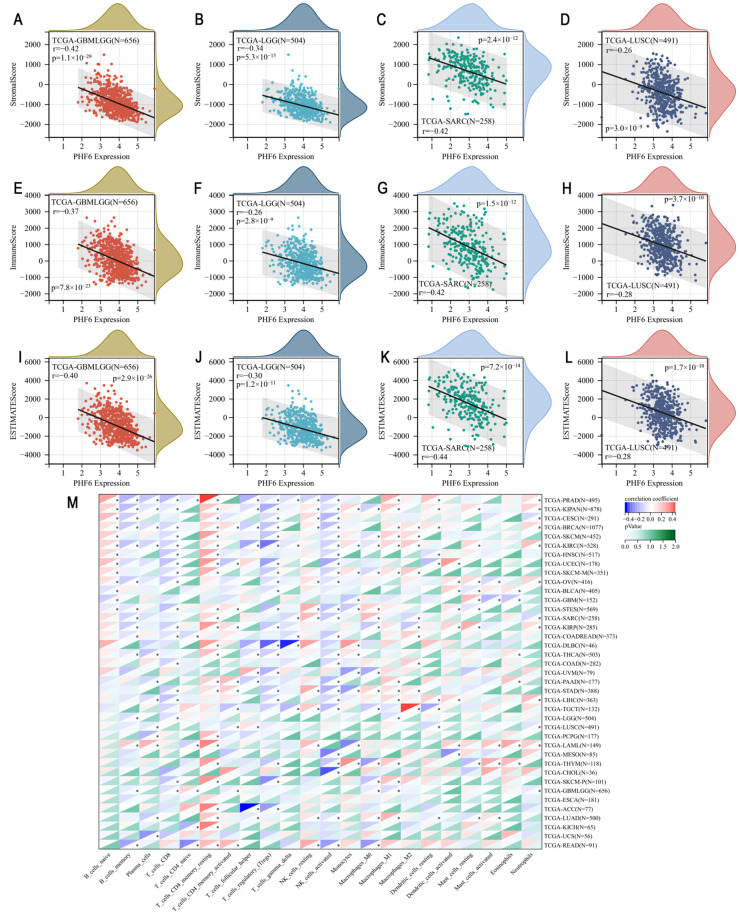
Tumor immune analysis in PHF6 expression. The correlations between PHF6 with stromal score (**A**–**D**), immune score (**E**–**H**), and ESTIMATE score (**I**–**L**) in GBMLGG, LGG, SARC, and LUSC. The correlation of PHF6 with immune infiltration levels based on CIBERSORT algorithm (**M**). * *p* < 0.05.

Utilizing the TCGA Pan-Cancer dataset, we calculated the immune infiltration score for each sample and analyzed the correlation between PHF6 and immune infiltration. The results showed that PHF6 was correlated with immune infiltration in 16 types of cancer (Table S1). Generally, PHF6 was negatively correlated with immune infiltration in most cancers. Specifically, PHF6 was negatively correlated with stromal score, immune score, and ESTIMATE score in glioma (Figure 3A,E,I), lower-grade glioma (LGG) (Figure 3B,F,J), sarcoma (SARC) (Figure 3C,G,K), and lung squamous cell carcinoma (LUSC) (Figure 3D,H,L). Immune cell infiltration varied with the expression level of PHF6 (Figure 3M). PHF6 was positively correlated with resting memory CD4+ T cells across many cancer types but negatively correlated with activated NK cells (Figure 3M). While the expression of PHF6 often indicated an immunosuppressive tumor microenvironment, characterized by increased immune cells associated with tumor suppression, some exceptions were observed. In cancers such as bladder urothelial carcinoma (BLCA), GBM, uveal melanoma (UVM), LGG, LUSC, mesothelioma (MESO), esophageal carcinoma (ESCA), and uterine carcinosarcoma (UCS), no significant relationship between PHF6 and tumor immune cell infiltration was found.

In addition, we explored the association between PHF6 and immune checkpoint genes in pan-cancer. We found that the expression of many immune checkpoint genes, either stimulatory or inhibitory, was positively correlated with PHF6 expression, such as CD276, EDNRB, VEGFA, HMGB1, BTN3A, TNFSF4, ENTPD1, and TLR4 (supplementary Figure S1). However, we found that a small number of stimulatory immune checkpoint genes were negatively correlated with PHF6 expression, such as TNFRSF18, TNFRSF4, CD70, CD27, GZMA, and CCL5 (supplementary Figure S1).

### 3.4. Enrichment Analysis

**Figure 4:**
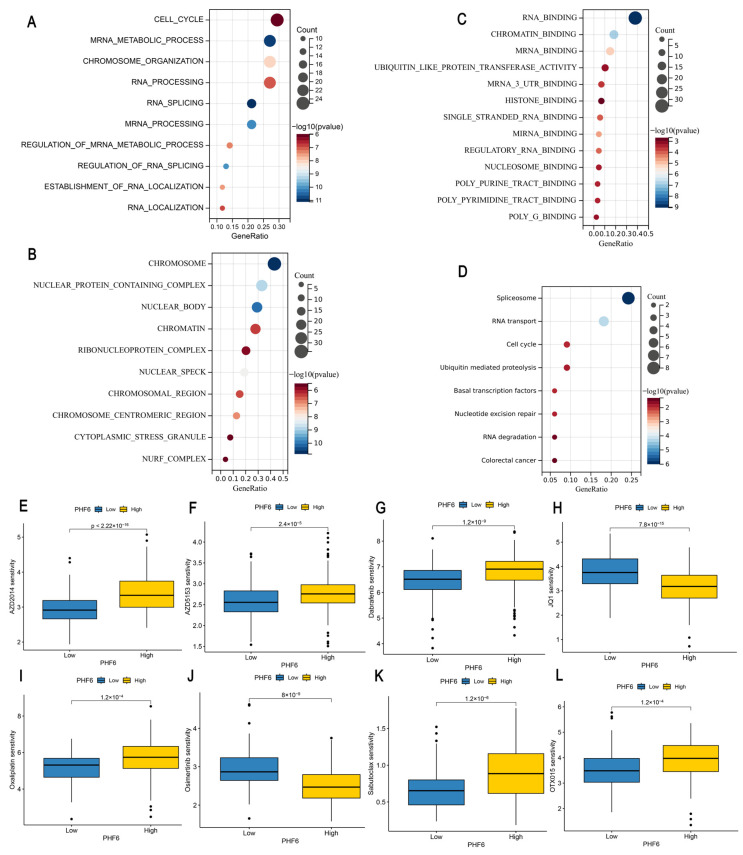
Enrichment analysis and drug sensitivity analysis of PHF6. GO analysis including biological processes (**A**), cellular components (**B**), and molecular functions (**C**) showed that PHF6 was enriched in cell cycle and chromatin-binding pathways (**A**–**C**). KEGG analysis indicated that PHF6 was related to spliceosome pathways (**D**). Impact of PHF6 on drug sensitivity in LIHC (**E**–**H**) and PAAD (**I**–**L**).

The top 100 genes significantly correlated with PHF6 were downloaded from the GEPIA database, and gene enrichment analysis was conducted to explore the potential molecular mechanisms of PHF6 in the occurrence and development of tumors. GO analysis showed that PHF6 was significantly related to cell cycle, mRNA metabolic progress, chromosome organization, chromatin, RNA binding, chromatin-binding, and histone-binding pathways (Figure 4A–C). KEGG analysis indicated that PHF6 was related to spliceosome, RNA transport, cell cycle, and ubiquitin mediated proteolysis pathways (Figure 4D). These results suggest that PHF6, as a chromatin-binding protein, may regulate gene transcription by interacting with chromatin, thereby influencing the occurrence and development of tumors. The drug sensitivity analysis indicated that PHF6 was closely related to tumor drug sensitivity, which could offer potential guidance and assistance for the precise treatment of tumors.

### 3.5. Drug Sensitivity Analysis

To further explore the relationship between PHF6 and anti-tumor drug sensitivity, we calculated the IC50 values of LIHC and PAAD samples based on the GDSC database. We found that in LIHC, the low PHF6 expression group was more sensitive to AZD2014 (Figure 4E), AZD5153 (Figure 4F), and dabrafenib (Figure 4G), whereas the high PHF6 expression group was more sensitive to JQ1 (Figure 4H). In PAAD, lower PHF6 expression levels made the samples more sensitive to oxaliplatin (Figure 4I) and sabutoclax (Figure 4K), whereas higher PHF6 expression increased sensitivity to Osimertinib (Figure 4J) and OTX015 (Figure 4L).

### 3.6. Correlation of PHF6 and Cancer Characteristics in Pan-Cancer

**Figure 5:**
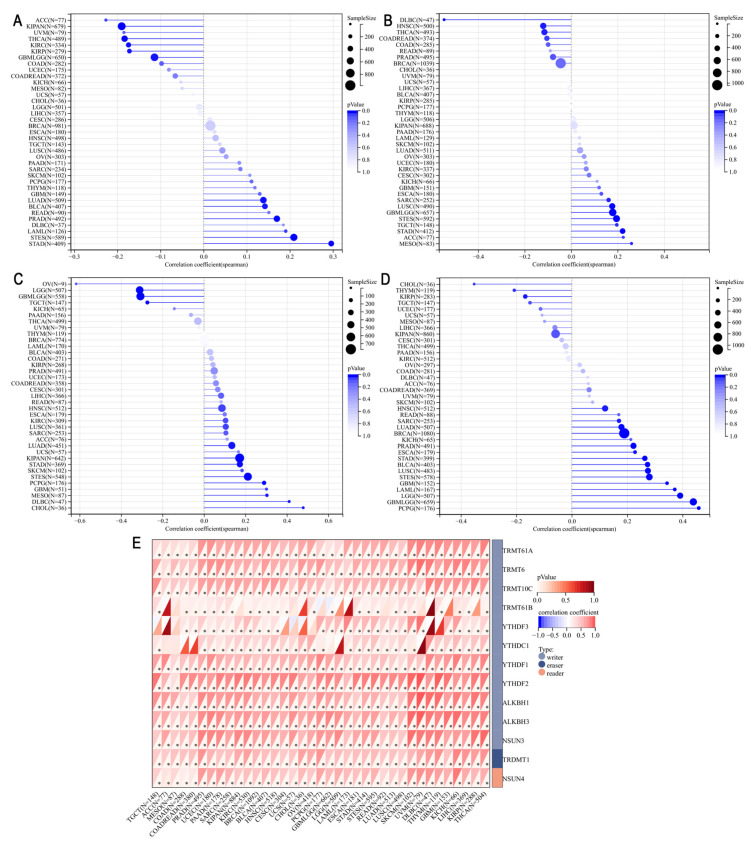
Correlations of PHF6 and TMB (**A**), microsatellite instability (**B**), DNAss (**C**), and RNAss (**D**). Correlations between PHF6 expression and RNA m1A modification (**E**). * *p* < 0.05.

We utilized data from the TCGA Pan-Cancer dataset to explore the correlation between PHF6 and tumor characteristics such as TMB, tumor stemness, and RNA methylation. Our findings revealed that PHF6 positively correlated with TMB in stomach adenocarcinoma (STAD), prostate adenocarcinoma (PRAD), stomach and esophageal carcinoma (STES), BLCA, and LUAD. Conversely, PHF6 was negatively correlated with TMB in THCA, KIRC, GBMLGG, and kidney renal papillary cell carcinoma (KIRP) (Figure 5A). PHF6 expression showed a negative correlation with MSI in lymphoid neoplasm diffuse large B-cell lymphoma (DLBC). However, PHF6 expression was positively correlated with MSI in MESO, ACC, and STAD (Figure 5B). We calculated correlation coefficients between PHF6 with DNAss and RNAss. Results indicated a positive correlation between PHF6 and DNAss in OV and a negative correlation in cholangiocarcinoma (CHOL) and DLBC (Figure 5C). PHF6 was positively correlated with RNAss in GBMLGG and PCPG (Figure 5D). Additionally, PHF6 expression was positively associated with the expression of RNA m1A modification-related genes in the majority of cancers (Figure 5E).

### 3.7. Knockout of PHF6 Suppresses the Malignant Phenotype of Liver Cancer and PAAD Cells

**Figure 6:**
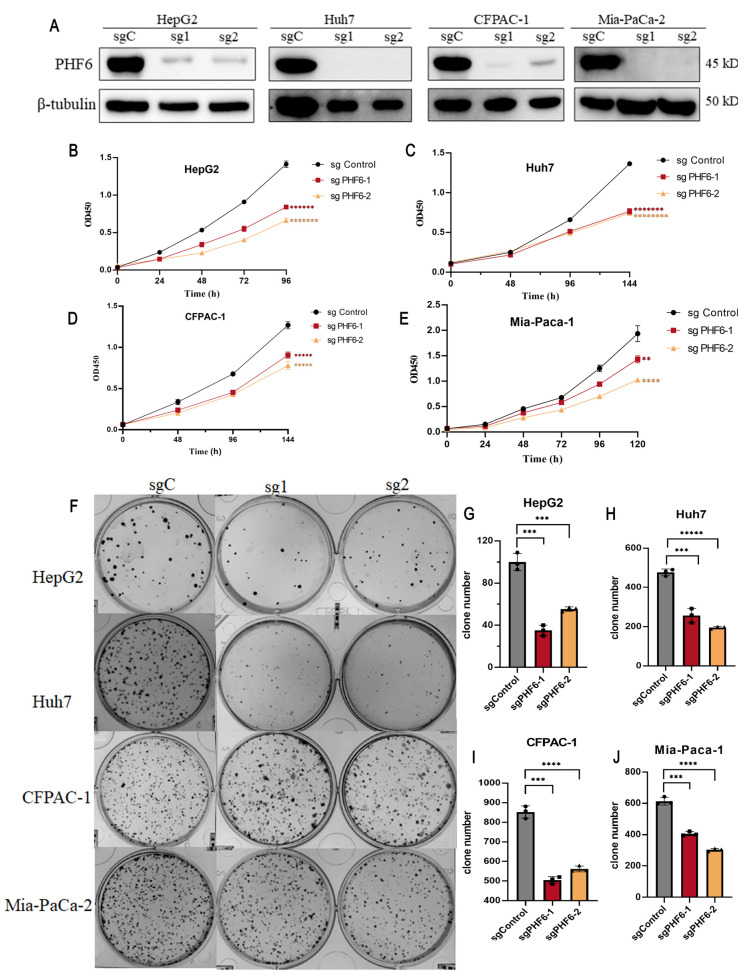
Knockout of PHF6 suppressed cell proliferation of liver cancer and PAAD cells. Western blot detected the knockout efficiency of PHF6 (**A**). PHF6 antibody (Santa Cruz Biotechnology, Inc., Dallas, TX, United States), β-tubulin (ZSGB-BIO, Beijing, China). sgC: sgControl, sg1: sgPHF6#, sg2: sgPHF6#2. The CCK-8 experiment on the proliferation of HepG2 (**B**), Huh7 (**C**), CFPAC-1 (**D**), and Mia-Paca-1 (**E**). Effect of PHF6 knockout colony formation (**F**) of HepG2 (**G**), Huh7 (**H**), CFPAC-1 (**I**), and Mia-Paca-1 (**J**). ** *p* < 0.01, *** *p* < 0.001, **** *p* < 0.0001, ***** *p* < 0.00001, ****** *p* < 0.000001, ******* *p* < 0.0000001, ******** *p* < 0.00000001.

The knockout of PHF6 in liver and pancreatic cancer cell lines was utilized to investigate the effect of PHF6 on cancer cell proliferation. Western blot analysis indicated that the knockout of PHF6 was effective (Figure 6A). CCK8 assay showed that depletion of PHF6 inhibited cell proliferation in both liver and pancreatic cancer cells (Figure 6B–E). The colony formation assays revealed that after PHF6 knockout, the number of colonies in liver cancer and PAAD cells was significantly reduced, and the proliferation ability of the cells was markedly impaired (Figure 6F–J). These results demonstrated that PHF6 played an important role in tumorigenesis of various cancers.

## 4. Discussion

PHF6 is a chromatin-binding protein located in the nucleus that regulates transcription. The PHF6 gene is situated on human chromosome Xq26.3, and mutations in this gene are known to cause BLFS [9,18]. PHF6 is expressed in nearly all tissues and is important for neurodevelopment and hematopoiesis. Knockout of PHF6 in hematopoietic stem cells could increase the self-renewal ability of cells and drive the development of blood cancer [19,20]. Therefore, PHF6 is generally regarded as having a tumor-suppressive function. In contrast, while PHF6 exhibits anti-tumor effects in T-ALL and AML, its overexpression in breast cancer [21] and other cancers promotes the proliferation of cancer cells, exhibiting a tumor-promoting role [22]. This suggests that distinct mechanisms of PHF6 are involved in tumorigenesis of various cancers. When PHF6 is inactivated by mutation, the early stages of normal blood development are disrupted. This disruption can synergize with the abnormal expression of genes such as TLX3, accelerating aberrant T-cell proliferation and advancing the progression of T-ALL [23]. However, PHF6 mutations are infrequent in solid tumors. PHF6 often collaborates with other molecules through elevated expression to activate oncogenic pathways, regulate epigenetics, and facilitate tumor growth. In breast cancer, PHF6 recruits the chromatin-remodeling factor BPTF for epigenetic modifications, thereby enhancing the transcriptional activity of the HIF pathway, which in turn promotes tumor angiogenesis and metabolic reprogramming [21]. We observed that PHF6 was significantly elevated in tumor tissues in most cancer types. High PHF6 expression was correlated with poor OS in LIHC, PAAD, and BRCA. Conversely, patients with high PHF6 expression experienced longer OS and DSS in GBMLGG, SKCM, and KIRC. These results highlight the complex role of PHF6 in tumorigenesis, suggesting it can act as either an oncogene or a tumor suppressor, necessitating careful exploration of its specific functions and mechanisms.

The tumor microenvironment (TME) consists of non-tumor cells and their secreted molecules within tumor tissues [24]. Immune cells in the TME primarily include T lymphocytes, myeloid-derived suppressor cells, tumor associated macrophages (TAMs), natural killer cells (NK), dendritic cells (DC), and neutrophils [25,26,27]. These immune cells interact with tumor cells by secreting cytokines or chemokines, potentially playing anti-tumor or pro-oncogenic roles under various conditions. Furthermore, interactions among different immune cells can lead to immune activation or suppression [28,29]. Our study found that PHF6 was negatively associated with immune infiltration in most cancers. The expression of PHF6 positively associated with resting-memory CD4+ T cells, while it was negatively correlated with activated NK cells across many cancer types. These results showed that PHF6 could be involved in regulating the TME, thereby promoting tumorigenesis. Common immune checkpoint molecules in tumors include PD-L1, PD-1, CTLA4, etc. PD-L1/PD-1 is the most prevalent immune checkpoint, playing a crucial role in regulating the TME and facilitating immune escape [30,31], with its expression predicting the efficacy of tumor immunotherapy [32,33]. Our study revealed that in certain tumors, PHF6 expression showed a significantly positive correlation with the expression of PD-L1/PD-1, CTLA4, HMGB1, TLR4, and other immune checkpoint genes, suggesting that PHF6 could contribute to immune escape by modulating these molecules. Studies have demonstrated that PHF6 R274X mutation alters the expression of key genes related to hematopoietic stem cell self-renewal and T cell activation [34]. Additionally, PHF6 knockout enhanced T cell migration by increasing IL32 production and secretion in endometrial cancer cells [35]. These findings underscore that PHF6 regulates the TME and might emerge as a target for tumor immunotherapy.

Pan-cancer tumor markers refer to molecular characteristics or biomarkers that guide treatment options across various cancers. The FDA has approved biomarkers for pan-cancer applications, including MSI and high TMB [36]. We found that PHF6 was positively correlated with TMB in STAD, STES, PRAD, BLCA, and LUAD. Additionally, PHF6 expression showed a positive correlation with MSI in MESO, ACC, and STAD. These results showed that PHF6 could serve as a potential biomarker for cancer therapy in these tumors. Furthermore, the relationship between PHF6 and tumor stemness was explored, demonstrating positive correlations between PHF6 and DNAss in OV and negative correlations in CHOL and DLBC. PHF6 expression also showed a positive correlation with RNAss in GBMLGG and PCPG. Thus, PHF6 may influence tumor stemness, thereby affecting tumor progression. Studies have shown that epigenetic changes, particularly DNA methylation and histone modification, function as molecular drivers of tumorigenesis alongside gene mutations, promoting the transformation of normal tissues into tumors ones [37,38]. Recent studies indicated that N1-methyladenosine (m1A) levels, m1A-related regulatory factors, and m1A-related RNA might represent novel biomarkers for cancer prognosis [39,40], and m1A regulators might offer breakthroughs in cancer treatment [41]. We observed that PHF6 was positively associated with the expression of RNA m1A machinery in most cancers, suggesting that PHF6 might influence tumor progression by modulating RNA m1A.

PHF6 is a versatile transcriptional regulator that can either inhibit or activate transcription initiation by interacting with the NuRD or UBF chromatin-remodeling complex. It also regulates transcriptional elongation in association with the PAF1 complex [3,42,43]. Previous studies have indicated that the deletion of PHF6, in the context of SMARCB1 deletion, disrupts the recruitment and stability of the remaining SWI/SNF complex members. This disruption leads to the loss of active chromatin in the promoter region and the stalling of RNA polymerase II progression [44]. Although our findings indicate that PHF6 knockout markedly slows tumor cell proliferation in liver and pancreatic cancer cells, the underlying mechanism requires further investigation.

In conclusion, bioinformatic analysis revealed that PHF6 expression was significantly higher in most cancer types. Moreover, PHF6 expression was strongly associated with prognosis, immune cell infiltration, and the expression of immune checkpoint genes in various cancers. Knockout of PHF6 inhibited the proliferation of liver cancer and pancreatic cancer cells. Therefore, we propose that PHF6 could potentially serve as a biomarker for guiding cancer diagnosis and treatment.

## Data Availability

The original data presented in the study are openly available in TCGA database at https://xenabrowser.net/ (accessed on 14 January 2025). Further inquiries can be directed to the corresponding author.

## Supplementary Materials

The following supporting information can be downloaded at: https://www.mdpi.com/article/10.3390/diagnostics16010110/s1, Figure S1: The correlation of PHF6 with immune checkpoint expression.; Table S1: The relationship between PHF6 and immune infiltration based on the Estimate.
