# Supplementary figures and images for: Systematic Pan-Cancer Analysis Identifies PHF6 as an Immunological and Prognostic Biomarker
Source: Diagnostics (Basel). 2025 Dec 29;16(1):110. doi: 10.3390/diagnostics16010110 (PMC12785632; doi:10.3390/diagnostics16010110)

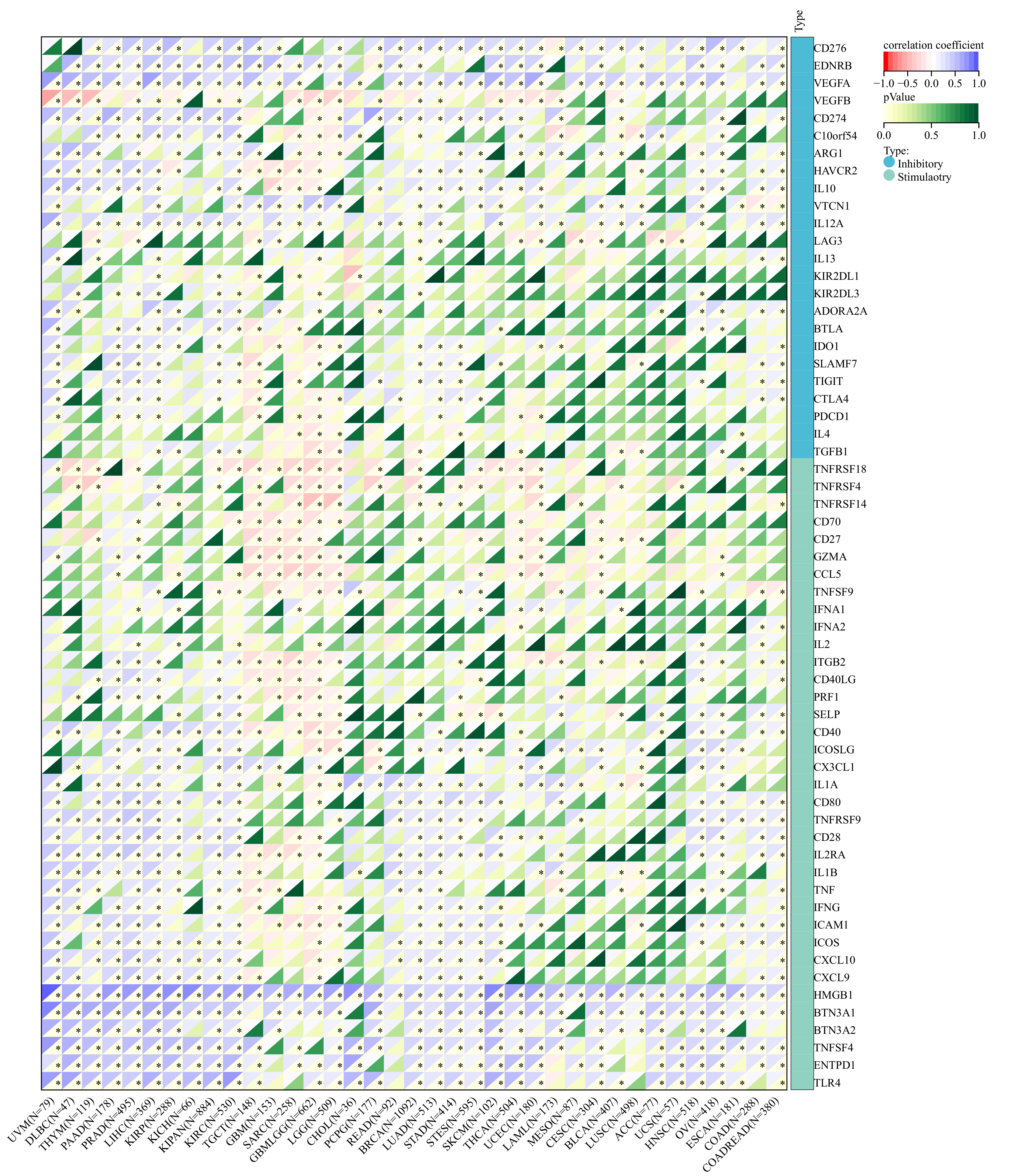

Supplement: Supplementary file 1 [file diagnostics-16-00110-s001.zip › Figure S1.tif]
